# Psychiatric Adverse Events and Administration Challenges Associated with GLP-1 Receptor Agonists for Weight Loss: A Real-World Analysis

**DOI:** 10.3390/ph19030365

**Published:** 2026-02-26

**Authors:** Ali Hindi, Mohamed Mekkawy, Hala Shokr

**Affiliations:** 1 Division of Pharmacy and Optometry, School of Health Sciences, Faculty of Biology, Medicine and Health, The University of Manchester, Manchester M13 9PL, UK; 2 Clinical Pharmacy Department, Alexandria Police Hospital, Alexandria 21511, Egypt; ph.m.mekkawy@gmail.com

**Keywords:** GLP-1 receptor agonists, psychiatric adverse events, liraglutide, semaglutide, tirzepatide, administration challenges

## Abstract

**Background**: Glucagon-like peptide-1 receptor agonists are increasingly prescribed for weight loss, but concerns remain regarding adverse events beyond gastrointestinal, renal, and pancreatic effects. Understanding these risks is essential to guide safe clinical application and public health policy. The study aims to characterize psychiatric risks, administration-related adverse events, and patterns of inappropriate use associated with semaglutide, liraglutide, and tirzepatide for weight management. **Methods:** Disproportionality analysis using proportional reporting ratios and reporting odds ratios was conducted to detect significant signals in adverse event reports within the U.S. Food and Drug Administration Adverse Event Reporting System, identifying semaglutide, liraglutide, or tirzepatide as drugs used for weight loss while excluding gastrointestinal, renal, and pancreatic adverse events. **Results:** Among 40,253 adverse event reports (68.6% female; median ages: semaglutide, 62 years; liraglutide, 59 years; tirzepatide, 53 years), semaglutide demonstrated the strongest disproportionality signal for psychiatric adverse events, notably anxiety (PRR 1.34, 95% CI 1.18–1.51), depression (PRR 1.83, 95% CI 1.62–2.07), and suicidal ideation (PRR 3.44, 95% CI 2.98–3.97). Tirzepatide showed markedly higher signals for injection-site reactions (PRR 7.98, 95% CI 7.8–8.18) and inappropriate use, including incorrect dosing and off-label administration (PRR 5.98, 95% CI 5.9–6.06). **Conclusions:** In real-world use, semaglutide is disproportionately associated with psychiatric adverse events, whereas tirzepatide demonstrates higher rates of injection-site complications and misuse. Liraglutide presents a comparatively lower risk profile. These findings underscore the need for vigilant psychiatric monitoring, patient education on injection technique and dosing, and stronger regulatory oversight to reduce misuse of GLP-1 receptor agonists for weight loss.

## 1. Introduction

Recently, glucagon-like peptide-1 receptor agonists (GLP-1 RAs) have become a cornerstone in the pharmacological management of obesity due to their dual impact on metabolic regulation and appetite suppression [1]. These agents mimic the action of endogenous GLP-1, enhancing insulin secretion, delaying gastric emptying, and reducing appetite [2]. Liraglutide and semaglutide, initially developed for glycaemic control in T2DM, were later approved by the U.S. Food and Drug Administration (FDA) for chronic weight management based on substantial weight reduction seen in clinical trials [3]. In November 2023, tirzepatide, a dual GLP-1 and glucose-dependent insulinotropic polypeptide (GIP) receptor agonist, was approved by the FDA for chronic weight management in adults with obesity or overweight [4].

The efficacy of these medications in achieving clinically significant weight loss has been demonstrated across several large randomized controlled trials, such as the STEP [5], SCALE [6], and SURMOUNT [7]. Compared to traditional weight management strategies such as lifestyle interventions or older pharmacotherapies, clinical data showed that patients using these injectables can lose up to 25.3% of their body weight compared to the 2–10% weight reduction typically achieved through conventional methods over the same period of time (68–72 weeks) [8,9,10]. These trials have also shown that GLP-1 RAs not only promote weight loss but also improve cardiometabolic risk profiles, contributing to their rapid adoption in both diabetes and obesity care [11,12]. As a result, their use has expanded significantly, not only among individuals with obesity and metabolic disease but also increasingly among younger and adults populations seeking weight loss despite no clinical indication [13]. This surge in demand has brought attention to their safety profile, particularly in real-world settings that extend beyond the controlled environment of clinical trials.

While gastrointestinal (GI) and pancreatic adverse events are well recognized and consistent with the pharmacodynamic properties of GLP-1 RAs, emerging post-marketing evidence has raised concerns about a broader range of potential adverse events [14]. These include psychiatric symptoms such as depression, anxiety, and suicidal ideation [15,16]; sexual dysfunction [17]; and other reported outcomes, including malignancy-related events [18,19]. Many of these safety signals have been highlighted through case reports, patient anecdotes, and social media discourse, prompting regulatory agencies and researchers to call for more comprehensive investigation [20,21,22]. However, the existing evidence remains inconclusive, as most RCTs are not designed or powered to detect rare, long-term, or unexpected adverse events [21,22].

Concerns regarding safety are further amplified by the increasing prevalence of inappropriate use of GLP-1 RAs, including use for cosmetic weight loss, use without appropriate medical supervision, and acquisition through non-regulated or online sources. This issue is particularly salient given the rapid expansion of GLP-1 RA use among younger populations; between 2020 and 2023, utilization among adolescents increased by 594%, with a disproportionately higher increase observed among females [13].

In parallel, injection site reactions including erythema, pain, swelling, and localized nodules have been reported with injectable GLP-1 therapies [23]. However, these reactions are underrepresented in clinical trial data, which typically prioritize systemic and serious adverse events, thereby underscoring the importance of real-world pharmacovigilance analyses.

The rapid expansion of GLP-1 RA use for weight management necessitates clearer characterization of their full adverse event spectrum. This is especially important for informing long-term treatment planning, identifying at-risk subgroups, and supporting patient-centered decision-making. Real-world pharmacovigilance data can play a critical role in this effort. The U.S. Food and Drug Administration’s Adverse Event Reporting System (FAERS) offers a large and publicly accessible dataset of spontaneously reported adverse drug events.

In this study, we aim to evaluate the adverse event profiles of semaglutide, liraglutide, and tirzepatide with a focus on less commonly investigated adverse events, specifically psychiatric disorders, injection site reactions, and other emerging areas of concern using FAERS data.

## 2. Results

**Figure 1:**
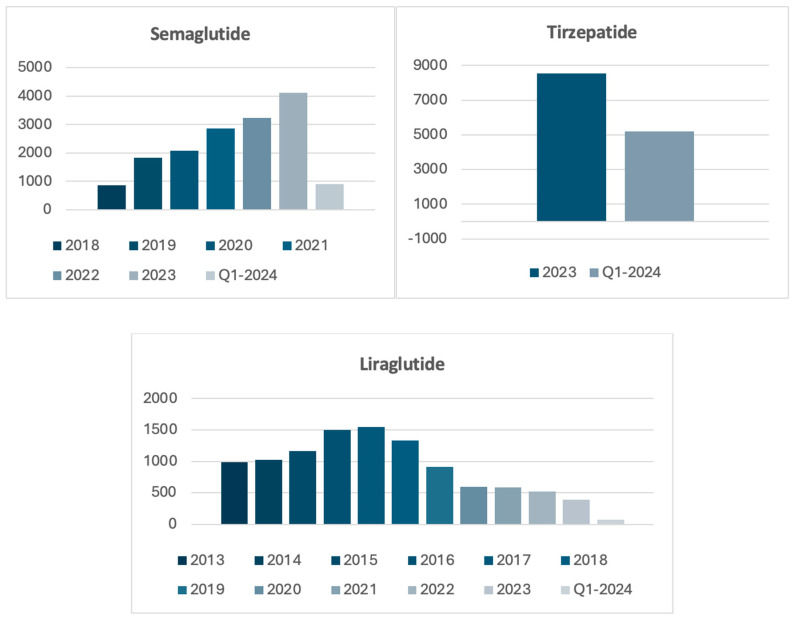
Reporting Years of Side Effects.

Inclusion periods for each drug were aligned with their respective FDA approval timelines for obesity: January 2013 to June 2024 for liraglutide, January 2018 to June 2024 for semaglutide, and January 2023 to June 2024 for tirzepatide (Figure 1).

During the study period, a total of 40,253 adverse drug reaction (ADR) reports were collected, with semaglutide accounting for 15,890 reports (39.5%), liraglutide for 10,623 (26.4%), and tirzepatide for 13,740 (34.1%). Across all three drugs, the majority of reports originated from females, who made up an average of 68.6% of the cases, while males accounted for 31.4%. In terms of geographic distribution, 85.3% of the reports were submitted from the United States. Consumers were the primary source of these reports, contributing to 74% of the total (Table 1).

Demographic data showed that the median weight of patients was 92.25 kg for semaglutide, 94 kg for liraglutide, and 92.5 kg for tirzepatide. The median age of patients varied slightly across the drugs: 62 years for semaglutide users, 59 years for those on liraglutide, and 53 years for tirzepatide.

The study identified 531 overall deaths associated with the use of these drugs, with 176 associated with semaglutide, 304 with liraglutide, and 51 with tirzepatide. Additionally, there were 438 life-threatening events reported: 217 for semaglutide, 195 for liraglutide, and 26 for tirzepatide. Hospitalisation was also a reported outcome in a subset of cases, occurring 2725 times for semaglutide, 2357 times for liraglutide, and 564 times for tirzepatide (Table 1).

### 2.1. Inappropriate Use

Across all three drugs, a total of 12,158 inappropriate use events were reported. Tirzepatide accounted for the majority of these cases (n = 9402), followed by semaglutide (n = 2020) and liraglutide (n = 736). Incorrect dosing was the most frequently reported inappropriate use event overall, representing 50.51% of tirzepatide cases (n = 4749), 12.47% of semaglutide cases (n = 252), and 8.28% of liraglutide cases (n = 61). Off-label use was also highly prevalent, comprising 77.42% (n = 1564) of semaglutide reports, 81.36% (n = 598) of liraglutide reports, and 21.29% (n = 2002) of tirzepatide reports. Tirzepatide also showed notable frequencies of extra dose administration (n = 1384; 14.72%), accidental underdose (n = 620; 6.59%), dose omission (n = 441; 4.69%), and accidental overdose (n = 206; 2.19%). Individual breakdown of the three drugs for inappropriate use is provided in Table 2.

### 2.2. Injection Site Reactions

A total of 6573 injection site reactions were reported: 5250 for tirzepatide, 685 for liraglutide, and 638 for semaglutide. Injection site pain was the most common reaction overall, particularly prominent in tirzepatide (n = 1872; 35.66%) and semaglutide (n = 219; 34.32%). Other frequently reported reactions across the three drugs included haemorrhage (tirzepatide: n = 970; 18.47%, semaglutide: n = 134; 21.00%), erythema (tirzepatide: n = 681; 12.97%, liraglutide: n = 140; 20.43%), and bruising (tirzepatide: n = 471; 8.97%, liraglutide: n = 125; 18.24%, semaglutide: n = 104; 16.30%). Less frequent but notable reactions included mass formation, swelling, injury, rash, and pruritus, with the highest overall incidence in the tirzepatide group. Individual breakdown of the three drugs for injection site reactions is provided in Table 2. Furthermore, tirzepatide exhibits striking figures across several categories. For extra dose administered, tirzepatide has 1384 associated reports out of 2955 reports, representing approximately 46.8% of total extra doses administered in the FAERS during that period. Similarly, for accidental underdose, tirzepatide shows 620 associated reports out of 1165 in total, accounting for 53.2%. Additionally, for injection site injury, tirzepatide has 211 associated reports out of 425 in total, equating to 49.6%. Notably, these high proportions were observed despite tirzepatide having a relatively short reporting period, from January 2023 to June 2024 (Table 2).

### 2.3. Psychiatric Events

A total of 2349 psychiatric adverse events were recorded: 1547 for semaglutide, 573 for liraglutide, and 229 for tirzepatide. Anxiety was the most commonly reported psychiatric event across all drugs, especially for tirzepatide (n = 95; 41.5%), followed by semaglutide (n = 262; 16.9%) and liraglutide (n = 76; 13.3%). Depression followed closely, comprising (n = 253; 16.35%) of semaglutide cases, (n = 83; 14.48%) of liraglutide cases, and (n = 59; 25.76%) of tirzepatide cases. Suicidal ideation was reported in 187 semaglutide cases (12.08%), 48 liraglutide cases (8.37%), and 41 tirzepatide cases (17.9%). Individual breakdown of the three drugs for psychiatric events is provided in Table 2.

### 2.4. Inferential Analysis

#### 2.4.1. Inappropriate Use

Tirzepatide demonstrated a significantly stronger disproportionality signal for overall inappropriate use (PRR: 5.98, ROR: 16.78) compared to liraglutide (PRR: 0.90, ROR: 0.89) and semaglutide (PRR: 1.28, ROR: 1.32). This pattern was consistent across subcategories including incorrect dose administered, extra dose administered, accidental underdose, off-label use, accidental overdose, and product dose omission. The non-overlapping confidence intervals suggest statistically significant differences in reporting patterns among the three drugs (Table 3).

#### 2.4.2. Injection Site Reaction

Tirzepatide also showed a stronger disproportionality signal for injection site reactions overall (PRR: 7.98; ROR: 12.3) compared to liraglutide (PRR: 1.39, ROR: 1.41) and semaglutide (PRR: 0.96, ROR: 0.96). This included specific events such as injection site pain, haemorrhage, bruising, mass, swelling, injury, rash, pruritus, and erythema (Table 3).

#### 2.4.3. Psychiatric

Semaglutide exhibited a modestly stronger disproportionality signal for psychiatric adverse events (PRR: 1.33, ROR: 1.36) compared to liraglutide (PRR: 0.67, ROR: 0.66) and tirzepatide (PRR: 0.61, ROR: 0.61). Psychiatric events included depression, suicidal ideation, anxiety, panic attack, and fatigue. No significant differences were observed among the three drugs in terms of emotional distress reports (Table 3).

## 3. Discussion

This study evaluated the safety profiles of semaglutide, liraglutide, and tirzepatide, focusing on psychiatric disorders, inappropriate use, and injection-site reactions. Our findings revealed distinct safety signals across the three drugs, with tirzepatide showing disproportionately higher reports of injection-site reactions and inappropriate use compared to semaglutide and liraglutide. In contrast, semaglutide showed a stronger association with psychiatric adverse events, although the degree of disproportionality was not substantially elevated.

The higher number of inappropriate use reports related to tirzepatide raises significant concerns about how individuals are accessing these medications and the adequacy of available information for consumers. Compounded versions of semaglutide and tirzepatide are widely available in the commercial market despite lacking FDA approval for weight loss. This situation has created a regulatory and clinical gray zone [24]. These compounded products are frequently used as alternatives during shortages and periods of high demand. However, they are not subject to FDA review for safety, efficacy, manufacturing standards, or dosing accuracy, thereby introducing substantial clinical risk [25,26]. As of 30 April 2025, the FDA had received 520 adverse event reports related to compounded semaglutide and 480 reports for compounded tirzepatide, highlighting both the widespread use and potential dangers of these unregulated alternatives [24].

While these figures are concerning on their own, our analysis of the FAERS database further revealed a disproportionately higher frequency of overdose reports involving tirzepatide compared to semaglutide and liraglutide.

Tirzepatide was introduced relatively recently and has demonstrated superior efficacy. This combination has driven extensive media coverage and marketing that frame it as a rapid weight loss solution. This public narrative may be driving individuals to seek unregulated or non-prescription sources, often without sufficient understanding of dosing requirements or safety risks. For instance, there has been cases of individuals accessing these medicines via online pharmacies, black markets, and beauty salons that typically offer little to no medical supervision and, in some cases, counterfeit medications [26,27]. The scale of the problem has prompted international warnings. Both the WHO and the UK Medicines and Healthcare Products Regulatory Agency (MHRA) have issued alerts regarding counterfeit weight loss injectables, citing severe adverse effects and, in some cases, fatalities [28,29].

Beyond misuse and counterfeit access, device-related adverse events emerged as a distinct and clinically relevant safety signal. We also observed a stronger disproportionality signal for injection-site reactions associated with tirzepatide compared to liraglutide and semaglutide, despite tirzepatide being relatively new to the market. Tirzepatide is administered via a multi-dose, disposable KwikPen, which features an integrated 29G, 5 mm fixed needle. This needle is hidden until the moment of injection, a design intended to reduce anxiety in needle-phobic patients [30]. However, real-world user feedback indicates that the plunger mechanism requires significantly more force than semaglutide or liraglutide pens, which are generally regarded as more intuitive and easier to use. This increased resistance may contribute to slower injections, greater user difficulty, and, in some cases, injection-site bruising or incomplete dose delivery [31]. Future improvements in device design such as reducing plunger resistance, enhancing ergonomic features, and incorporating clearer user feedback mechanisms may help minimize injection site reactions and improve overall patient experience.

Beyond dosing challenges, cutaneous adverse effects are an important and increasingly recognized concern in GLP-1RA therapy [32,33]. Our analysis of the FAERS database revealed that tirzepatide accounts for nearly half of the total reports related to extra doses administered, accidental underdosing, and injection site injuries in the system. This disproportionate representation suggests that administration difficulties and device-related factors may significantly contribute to the observed safety signals. Similarly, recent reviews of large-scale clinical trials identified rash, erythema, and pruritus as the most commonly reported injection-site reactions among users [33,34]. These reactions occur more frequently with tirzepatide compared to liraglutide and semaglutide, which tend to have lower injection site reaction rates [34,35]. Notably, findings from the SURPASS clinical program, a key body of evidence evaluating GLP-1 drugs, demonstrated a dose-dependent increase in injection-site reactions, particularly at higher doses (10 mg and 15 mg) [36,37]. This trend suggests that a localized accumulation of the drug may contribute to injection site reaction frequency, reinforcing the need for patient education on proper injection technique and site rotation. In addition, the European/UK version of the pen includes a rotational dose-selector mechanism, which can allow for a “fifth dose” beyond the labeled four doses per pen. While intended to reduce waste, this feature introduces ambiguity about proper usage and may inadvertently contribute to dosing errors or intentional overuse, as also reflected in FAERS narratives [38]. In contrast, semaglutide and liraglutide use more intuitive prefilled pens with simpler injection mechanics and standardized needle attachments, offering greater user familiarity and fewer mechanical issues [39]. While these findings primarily relate to administration and device factors, our analysis also identified signals that extend beyond mechanical explanations.

We next examined psychiatric adverse events associated with GLP-1 receptor agonists. Compared to previous pharmacovigilance analyses such as the EudraVigilance review (2021–2023), our dataset showed a notably higher proportion of psychiatric reports overall, particularly for semaglutide and tirzepatide. While depression was the most frequently reported event in the EudraVigilance data, anxiety featured more prominently in our findings, especially for tirzepatide [40]. In addition, our findings indicate a proportionally higher reporting of suicidal ideation associated with semaglutide compared to liraglutide and tirzepatide. This emerging signal aligns with recent pharmacovigilance analyses, including a disproportionality study using the World Health Organization’s global database, which identified a potential association between semaglutide and suicidal ideation, an observation that warrants further research [41]. Although these findings generate important safety hypotheses, they must be interpreted within the methodological constraints of spontaneous reporting systems.

This study provides a comparative safety analysis of injection site reactions, inappropriate use, and psychiatric adverse events for GLP-1RAs. Several limitations inherent to the analysis of spontaneous reporting systems must be acknowledged. First, passive surveillance systems introduce well-established biases, including underreporting, overreporting, incomplete information, and reporting delays. These factors may influence the strength and detection of the safety signals we identified. Second, and critically, the absence of a defined base population precludes the calculation of incidence rates or relative risks. Consequently, our findings indicate disproportionality of reporting rather than definitive causality. Third, as tirzepatide received regulatory approval more recently than liraglutide and semaglutide, its safety profile is subject to the Weber effect (i.e., increased reporting frequency immediately after a drug’s launch). This may lead to an artificial inflation of signals for tirzepatide compared to the older drugs. Furthermore, this study did not adjust for potential confounding factors, such as underlying patient comorbidities, disease severity, specific drug dosages, or the use of concomitant medications. These unmeasured variables could influence the observed associations. Despite these limitations, the FAERS remains an invaluable tool for generating hypotheses regarding post-marketing drug safety. Unlike RCTs, which are conducted in highly selected populations under controlled conditions, FAERS provides insight into real-world drug use and can detect rare or long-term adverse events that may not be evident in pre-marketing studies. The signals identified here warrant further investigation through formal epidemiological studies designed to overcome these limitations. Education campaigns should clearly inform patients on proper dosing and administration; stricter regulatory oversight is crucial to address compounded and counterfeit GLP-1 receptor agonists; and collaboration between healthcare providers, regulators, and pharmaceutical companies is needed to mitigate misuse.

## 4. Materials and Methods

This study utilized a retrospective, observational pharmacovigilance design based on data from the U.S. FAERS data. FAERS data were extracted from the publicly available Quarterly Data Extract Files, covering the period from January 2018 to June 2024. The analysis focused on three GLP-1 receptor agonists, namely semaglutide, liraglutide, and tirzepatide, specifically in the context of weight management.

Data was downloaded as ASCII files from the FDA official website and then extracted, combined, and queried using DB Browser for SQLite Version 3.13.1. For each query, datasets were cleaned to remove duplicate entries, identified through matching case IDs, patient demographics, event dates, and drug names, retaining only the most recent version of each case following FDA guidance on deduplication.

We included reports that identified one of the three drugs as a suspect agent and contained an adverse event falling outside the gastrointestinal, renal, and pancreatic domains. Specifically, the study focused on adverse events related to psychiatric conditions, injection site reactions, and other emerging events such as fatigue and hypersensitivity. Reports with missing essential information (e.g., age, gender, suspect drug) were excluded from analysis to maintain data integrity. Drug names were standardized using generic names according to the FDA’s Structured Product Labeling resources, and AEs were coded using MedDRA version 27.0 Preferred Terms (PTs). Higher-level clinical categories were constructed from manually selected PTs based on predefined clinical relevance rather than Standardised MedDRA Queries.

To identify potential safety signals, disproportionality analyses were conducted using two standard methods: the Proportional Reporting Ratio (PRR) and the Reporting Odds Ratio (ROR). These metrics compared the frequency of specific adverse events reported for a drug of interest to the frequency of those events for all other drugs in the FAERS database. PRR and ROR were calculated as follows:

$$PRR=\frac{a/(a\;+\;b)}{c/(c\;+\;d)}\;\;\;\;\;\;\;\mathrm{ROR}=\frac{a/b}{c/d}$$

where a represents the number of reports for the event of interest with the drug, b the number of reports for other events with the drug, c the number of reports for the same event with other drugs, and d all other reports in the database. We considered a signal present when the 95% confidence interval of the PRR or ROR excluded 1.0, indicating a statistically significant increase in reporting frequency [42,43]. Signal strength across the three drugs was assessed using descriptive ranking rather than formal statistical comparisons between agents.

Data management and analysis were performed using R (Version 4.4.2) and Python (Version 3.13.0), following FDA-recommended practices for FAERS data handling. As the analysis was conducted using de-identified, publicly available data, institutional review board (IRB) approval was not required.

## 5. Conclusions

Our study leveraging the FAERS database provides a systematic comparison of the safety profiles of semaglutide, liraglutide, and tirzepatide. While previous concerns have focused on psychiatric adverse events, our real-world analysis found a less pronounced safety signal in this domain. In contrast, we identified robust and disproportionate reporting for inappropriate use, misuse, and injection site reactions, with tirzepatide demonstrating a notably stronger association compared to the other agents. The strong signal for injection site reactions with tirzepatide warrants heightened vigilance to ensure proper administration. Similarly, the signal for misuse highlights an emerging public health concern that requires regulatory and clinical attention. Furthermore, the strong signal concerning misuse and inappropriate use underscores an emerging public health risk that warrants immediate attention. Future initiatives should focus on two key areas: first, the development of pharmacovigilance strategies and public health campaigns to mitigate the risks of misuse and educate on safe use. Second, further research is needed to understand the behavioral drivers and circumstances behind the inappropriate use of these medications.

## Figures and Tables

**Table 1:** Study Population Characteristics.

Characteristics	Semaglutide-Induced Overall AEs (n = 15,890)	Liraglutide-Induced Overall AEs (n = 10,623)	Tirzepatide-Induced Overall AEs (n = 13,740)
**Gender, No. (%)**	15,620 (98.3%)	10,525 (99.1%)	12,700 (92.4%)
Female	9891 (63.3%)	6971 (66.2%)	9699 (76.4%)
Male	5729 (36.7%)	3554 (33.8%)	3001 (23.6%)
**Age (years), No. (%)**	12,438 (78.3%)	8630 (81.2%)	11,770 (85.7%)
<18	50 (0.4%)	37 (0.4%)	1 (0.0%)
18–65	7507 (60.4%)	5975 (69.3%)	9760 (82.9%)
>65	4881 (39.2%)	2618 (30.3%)	2009 (17.1%)
**Median (years)**	62	59	53
**Weight (kg), No. (%)**	4098 (25.8%)	3640 (34.3%)	620 (45.1%)
<80	1132 (27.6%)	938 (25.8%)	189 (30.5%)
80–100	1456 (35.5%)	1269 (34.9%)	202 (32.6%)
>100	1510 (36.9%)	1433 (39.3%)	229 (36.9%)
**Median weight (kg)**	92.25	94	92.5
**Reporting countries, No. (%)**	14,403 (90.6%)	9911 (93.3%)	13,282 (96.7%)
US	11,812 (82%)	7534 (76%)	13,003 (97.9%)
Non-US	2591 (18%)	2377 (24%)	279 (2.1%)
**Outcomes, No. (%)**	7624 (48%)	5943 (55.9%)	1325 (9.6%)
Death	176 (2.3%)	304 (5.1%)	51 (3.8%)
Life-threatening	217 (2.8%)	195 (3.3%)	26 (2%)
Hospitalization	2725 (35.7%)	2357 (39.7%)	564 (42.6%)
Disability	318 (4.2%)	115 (1.9%)	45 (3.4%)
Congenital Anomaly	9 (0.1%)	13 (0.2%)	2 (0.2%)
Other outcomes	4026 (52.9%)	2933 (49.4)	610 (46%)
Required Intervention to Prevent Permanent Impairment/Damage	153 (2%)	26 (0.4%)	27 (2%)
**Reporters, No. (%)**	15,790 (99.4%)	10,463 (98.5%)	13,717 (99.8%)
Health professional	5098 (32.3%)	4242 (40.5%)	702 (5.1%)
Consumer	10,692 (67.7%)	6221 (59.5%)	13,015 (94.9%)

**Table 2:** Full dataset of main adverse event and inappropriate use reports related to the use of tirzepatide, semaglutide, and liraglutide.

	Semaglutide ^a^		Liraglutide ^b^		Tirzepatide ^c^	
Side Effect	Total No. of Reports January 2018–June 2024	No. of Reports Associated with the Drug	Total No. of Reports January 2013–June 2024	No. of Reports Associated with the Drug	Total No. of Reports January 2023–June 2024	No. of Reports Associated with the Drug
**Inappropriate use (all)**	373,116	2020	507,338	736	80,604	9402
Incorrect dose administered	63,074	252	94,559	61	15,747	4749
Extra dose administered	9406	94	14,345	44	2955	1384
Accidental underdose	3634	1	4181	2	1165	620
Off label use	190,735	1564	280,557	598	34,123	2002
Accidental overdose	5933	25	10,929	14	1207	206
Product dose omission issue	100,334	84	102,767	17	25,407	441
**Injection site reaction (all)**	156,665	638	305,093	685	35,046	5250
Injection site pain	56,833	219	106,595	115	13,416	1872
Injection site haemorrhage	20,256	134	33,236	44	4442	970
Injection site bruising	14,767	104	31,253	125	3110	471
Injection site mass	9821	62	16,327	54	1756	267
Injection site swelling	15,097	39	30,189	30	3525	231
Injection site injury	1439	10	1944	5	425	211
Injection site rash	5940	4	11,641	56	1487	189
Injection site pruritus	12,193	9	27,159	116	2498	358
Injection site erythema	20,319	57	46,749	140	4387	681
**Psychiatric (all)**	276,345	1547	525,016	573	14,741	229
Depression	32,913	253	71,195	83	4175	59
Suicidal ideation	13,023	187	25,331	48	2391	41
Anxiety	46,520	262	92,297	76	6517	95
Panic attack	6652	56	12,735	13	1145	31
Emotional distress	5952	14	15,819	1	513	3
Fatigue	171,285	775	307,639	352	-	-

(a) Semaglutide. Search key words: semaglutide, ozempic, rybelsus, wegovy; Total number of reports in the database: 3,763,146; Total number of drug reports: 15,890. (b) Liraglutide. Search key words: Liraglutide, Victoza, Saxenda; Total number of reports in the database: 6,560,878; Total number of drug reports: 10,623. (c) Tirzapetide. Search key words: Tirzepatide, Mounjaro, Zepbound; Total number of reports in the database: 636,362; Total number of drug reports: 13,740.

**Table 3:** Comparative Analysis of proportional reporting ratios and reporting odds ratios for reported adverse drug reactions and inappropriate use.

	Semaglutide		Liraglutide		Tirzepatide		Relative Signal Strength *
Side Effect	PRR (95% CI)	ROR (95% CI)	PRR (95% CI)	ROR (95% CI)	PRR (95% CI)	ROR (95% CI)	
**Inappropriate use (all)**	1.28 (1.23–1.34)	1.32 (1.26–1.39)	0.9 (0.84–0.96)	0.89 (0.82–0.96)	5.98 (5.9–6.06)	16.78 (16.18–17.4)	PRR: T > S > L ROR: T > S > L
Incorrect dose administered	0.95 (0.84–1.07)	0.95 (0.83–1.07)	0.4 (0.31–0.51)	0.39 (0.31–0.51)	19.57 (19–20.15)	29.37 (28.2–30.57)	PRR: T > S > L ROR: T > S > L
Extra dose administered	2.38 (1.94–2.92)	2.39 (1.95–2.93)	1.9 (1.41–2.55)	1.9 (1.41–2.56)	39.92 (37.2–42.8)	44.28 (41.1–47.7)	PRR: T > S = L PRR: T > S = L
Accidental underdose	0.06 (0.01–0.46)	0.06 (0.01–0.46)	0.3 (0.07–1.18)	0.29 (0.07–1.18)	51.55 (46–57.77)	53.94 (48.01–60.6)	PRR: T > S = L ROR: T > S = L
Off label use	1.95 (1.86–2.04)	2.05 (1.95–2.16)	1.32 (1.22–1.42)	1.34 (1.23–1.45)	2.82 (2.71–2.95)	3.14 (2.99–3.29)	PRR: T > S > L ROR: T > S > L
Accidental overdose	1 (0.67–1.48)	1 (0.67–1.48)	0.79 (0.47–1.34)	0.79 (0.47–1.34)	9.33 (8.03–10.8)	9.45 (8.13–10.99)	PRR: T > S = L ROR: T > S = L
Product dose omission issue	0.2 (0.16–0.24)	0.19 (0.16–0.24)	0.1 (0.06–0.16)	0.1 (0.06–0.16)	0.8 (0.73–0.88)	0.79 (0.72–0.87)	PRR: T > S = L ROR: T > S = L
**Injection site reaction (all)**	0.96 (0.89–1.04)	0.96 (0.89–1.04)	1.39 (1.29–1.49)	1.41 (1.31–1.53)	7.98 (7.8–8.18)	12.3 (11.86–12.8)	PRR: T > L > S ROR: T > L > S
Injection site pain	0.91 (0.8–1.04)	0.91 (0.8–1.04)	0.67 (0.56–0.8)	0.66 (0.55–0.8)	7.35 (7.02–7.69)	8.35 (7.93–8.8)	PRR: T > S = L ROR: T > S = L
Injection site haemorrhage	1.57 (1.33–1.86)	1.58 (1.33–1.87)	0.82 (0.61–1.1)	0.82 (0.61–1.1)	12.66 (11.8–13.6)	13.55 (12.59–14.6)	PRR: T > S > L ROR: T > S > L
Injection site bruising	1.67 (1.38–2.03)	1.68 (1.38–2.04)	2.48 (2.08–2.95)	2.49 (2.09–2.98)	8.09 ( 7.34–8.91)	8.34 (7.55–9.21)	PRR: T > L > S ROR: T > L > S
Injection site mass	1.5 (1.17–1.92)	1.5 (1.17–1.93)	2.05 (1.57–2.67)	2.05 (1.57–2.68)	8.13 (7.14–9.25)	8.27 (7.25–9.43)	PRR: T > L > S ROR: T > L > S
Injection site swelling	0.61 (0.45–0.84)	0.61 (0.45–0.84)	0.61 (0.43–0.88)	0.61 (0.43–0.88)	3.18 (2.78–3.63)	3.22 (2.81–3.68)	PRR: T > S = L ROR: T > S = L
Injection site injury	1.65 (0.89–3.07)	1.65 (0.89–3.08)	1.59 (0.66–3.82)	1.59 (0.66–3.83)	44.68 (36.97–54)	45.36 (37.48–54.9)	PRR: T > S = L ROR: T > S = L
Injection site rash	0.16 (0.06–0.42)	0.16 (0.06–0.42)	2.98 (2.29–3.87)	2.99 (2.3–3.89)	6.6 (5.67–7.68)	6.68 (5.73–7.78)	PRR: T > L > S ROR: T > L > S
Injection site pruritus	0.17 (0.09–0.33)	0.17 (0.09–0.33)	2.64 (2.21–3.17)	2.66 (2.22–3.2)	7.58 (6.79–8.47)	7.76 (6.93–8.69)	PRR: T > L > S ROR: T > L > S
Injection site erythema	0.66 (0.51–0.86)	0.66 (0.51–0.86)	1.85 (1.57–2.18)	1.86 (1.58–2.2)	8.33 (7.69–9.02)	8.71 (8.01–9.47)	PRR: T > L > S ROR: T > L > S
**Psychiatric (all)**	1.33 (1.27–1.39)	1.36 (1.29–1.44)	0.67 (0.62–0.73)	0.66 (0.6–0.71)	0.61 (0.56–0.67)	0.6 (0.55–0.65)	PRR: S > L = T ROR: S > L = T
Depression	1.83 (1.62–2.07)	1.84 (1.62–2.08)	0.72 (0.58–0.89)	0.72 (0.58–0.89)	0.65 (0.5–0.84)	0.65 (0.5–0.84)	PRR: S > L = T ROR: S > L = T
Suicidal Ideation	3.44 (2.98–3.97)	3.46 (3–4.01)	1.17 (0.88–1.55)	1.17 (0.88–1.56)	0.79 (0.58–1.08)	0.79 (0.58–1.08)	PRR: S > L = T ROR: S > L = T
Anxiety	1.34 (1.18–1.51)	1.34 (1.19–1.52)	0.51 (0.41–0.64)	0.5 (0.4–0.63)	0.67 (0.55–0.82)	0.67 (0.55–0.82)	PRR: S > L = T ROR: S > L = T
Panic attack	2 (1.54–2.6)	2.01 (1.54–2.61)	0.63 (0.37–1.09)	0.63 (0.37–1.09)	1.26 (0.88–1.8)	1.26 (0.88–1.8)	PRR: S > L = T ROR: S > L = T
Emotional distress	0.56 (0.33–0.94)	0.56 (0.33–0.94)	0.04 (0.01–0.28)	0.04 (0.01–0.28)	0.27 (0.09–0.83)	0.27 (0.09–0.83)	PRR: S = L = T ROR: S = L = T
Fatigue	1.07 (1–1.15)	1.08 (1–1.16)	0.71 (0.64–0.78)	0.7 (0.63–0.77)	0.55 (0.5–0.62)	0.54 (0.49–0.61)	PRR: S > L > T ROR: S > L > T

PRR: proportional reporting ratio; ROR: reporting odds ratio; CI: confidence interval; S: Semaglutide; L: Liraglutide; T: Tirzepatide. * Descriptive ranking of signal strength.

## Data Availability

The original contributions presented in this study are included in the article. Further inquiries can be directed to the corresponding authors.
